# Decreased frequency of sugar-sweetened beverages intake among young children following the implementation of the health promotion levy in South Africa

**DOI:** 10.1017/S1368980024002623

**Published:** 2025-01-07

**Authors:** H Salome Kruger, Tertia van Zyl, Makama A Monyeki, Cristian Ricci, Ruan Kruger

**Affiliations:** 1 Centre of Excellence for Nutrition, North-West University, Potchefstroom, South Africa; 2 Medical Research Council Research Unit for Hypertension and Cardiovascular Disease, North-West University, Potchefstroom, South Africa; 3 Physical Activity, Sport and Recreation Research Focus Area, North-West University, Potchefstroom, South Africa; 4 Africa Unit for Trans-disciplinary Health Research, North-West University, Potchefstroom, South Africa; 5 Hypertension in Africa Research Team, North-West University, Potchefstroom, South Africa

**Keywords:** Sugar-sweetened beverages, Sugar tax, Children, South Africa

## Abstract

**Objective:**

This study assessed the association between baseline sociodemographic variables, body composition and 4-year changes in the intake of food groups, including sugar-sweetened beverages (SSB) among children, after the implementation of the health promotion levy.

**Design:**

Prospective cohort study.

**Setting:**

Ten schools in North West Province, South Africa.

**Participants:**

950 children aged 5–9 years at baseline and data of 672 children at follow-up. The frequency of intake from healthy and unhealthy food groups was assessed by questionnaire. Anthropometric and sociodemographic information were collected; BMI-for-age z-scores were calculated. The health promotion levy was implemented after baseline and follow-up measurements were done after 4 years. A random intercept generalised linear mixed model analysis was applied to investigate the time effect of the weekly intake of the foods adjusting for BMI-for-age z-scores and sociodemographic characteristics of the children.

**Results:**

The weekly frequency of intake from most food groups remained unchanged at follow-up. The frequency of SSB intake decreased significantly over 4 years. Decreased intake of SSB was not linked to increased intake of healthy foods. Changes in intakes from SSB were not associated with household income, parental education or BMI-for-age z-score categories. A decreased frequency of intake from SSB was observed following the implementation of the health promotion levy after baseline, in line with reports of national decreases in SSB sales in South Africa since 2017.

**Conclusions:**

The decreased frequency of SSB intakes following the implementation of the health promotion levy in South Africa may indicate that health policies can promote healthier dietary habits.

Obesity is a global public health problem among children and adolescents^(1)^. The increasing prevalence of paediatric overweight and obesity in Africa has been linked to increasing access to energy-dense and processed foods, as well as decreasing physical activity^(2)^. A recent study in 5- to 9-year-old South African children revealed a prevalence of 15 % overweight and 4 % obesity^(3)^. Unhealthy dietary behaviours during childhood may result in an increased risk for adult obesity, related non-communicable disease and early-onset CVD^(4)^. Energy-dense diets throughout childhood are linked to obesity^(5)^, and a meta-analysis of studies in children indicated that sugar-sweetened beverages (SSB) consumption may increase markers of obesity^(6)^. Intake of SSB has been targeted for intervention programmes aimed at children in several countries^(7,8)^. A sugar tax or levy on SSB at the point of sale is considered as an effective way to decrease the sales^(9)^ and intakes of SSB^(10)^, as well as the prevalence of overweight and obesity^(11)^. These reports were based on national sales data from the manufacturing industry^(9)^, household surveys and sales at shops and vending machines, including the results of a meta-analysis with data from a range of low- and high-income countries^(10,11)^. Most studies from the meta-analysis on the link between SSB sales and obesity indicators were performed among adults in the USA^(11)^, with one study among US children and adolescents^(12)^. A health promotion levy on cold SSB at the point of sale was implemented in South Africa in 2018 through the Rates and Monetary Amounts and Amendment of Revenue Laws Act, 2017 – Act No. 14 of 2017. This levy is a form of excise tax only on sugar-sweetened cold drinks, fixed at 2·1 South African cent per gram of the sugar content exceeding 4 g per 100 ml, whereas the first 4 g per 100 ml are levy free^(13)^.

Limited data are available on the intakes of SSB of South African children of primary school age, and to our knowledge, no reports of changes in cold SSB intakes from a longitudinal study after the implementation of the health promotion levy are available. Therefore, the aim of this part of the study was to assess the association between baseline sociodemographic variables, body composition and 4-year changes in intake from healthy and unhealthy food groups, including SSB, among children in the North West Province, South Africa, after the implementation of the health promotion levy. This study is part of a larger longitudinal study aimed to determine whether the exposure to higher adiposity, together with lifestyle factors, increases the odds of having elevated blood pressure^(14)^. The health promotion levy was implemented after baseline measurements had been completed, and the follow-up 4 years after baseline provided an opportunity to evaluate the tax by using an ongoing study.

## Methodology

This study is part of the longitudinal Exercise, Arterial Modulation and Nutrition in Youth South Africa (ExAMIN Youth SA) study among 5- to 9-year-old children, aimed to determine the prevalence of childhood hypertension and obesity^(3,14)^. The study protocol was registered in a clinical trials registry (ClinicalTrials.gov Identifier: NCT04056377), according to the Standard Protocol Items: Recommendations for Interventional Trials guidelines^(15)^.

### Assessment of sample size adequacy

The research team invited representatives of urban schools from the Dr Kenneth Kaunda District in the North West province, South Africa, to participate in this study. Baseline data were collected from 1103 children from ten participating schools during 2017–2018. The sample size adequacy was performed considering the type-III test on the time coefficient of the mixed model investigating the specific food intake change over time. To this aim, we consider a standardised effect size F of 0·15 corresponding to a medium to small standardised effect size and a type-I error rate of 5 %. In this condition, a sample size above 800 children is sufficient to provide a type-II error risk below 20 % (power above 80 %). The G * Power version 9.1.7 has been used to perform the power calculations. After accounting for dropouts, incomplete questionnaires or school absentees, a total of 950 children with complete baseline data were included in the analysis for this part of the study.

### Population and setting

The Department of Education and school principals from the included schools gave permission for participation in this research project. Quintile groups are applied to South African schools according to the employment rate and literacy of adults in the relevant living area to determine government funding of schools. Quintile 1 represents the schools in areas with the highest unemployment, whereas Quintile 5 schools are in areas with the lowest unemployment and highest literacy^(16)^. Participating schools from two municipalities, 50 km apart, spanned quintiles 3–5 (Municipality A: Q3 = 2, Q4 = 2, Q5 = 1; Municipality B: Q3 = 3, Q4 = 1, Q5 = 1^(3,14)^.

**Fig. 1:**
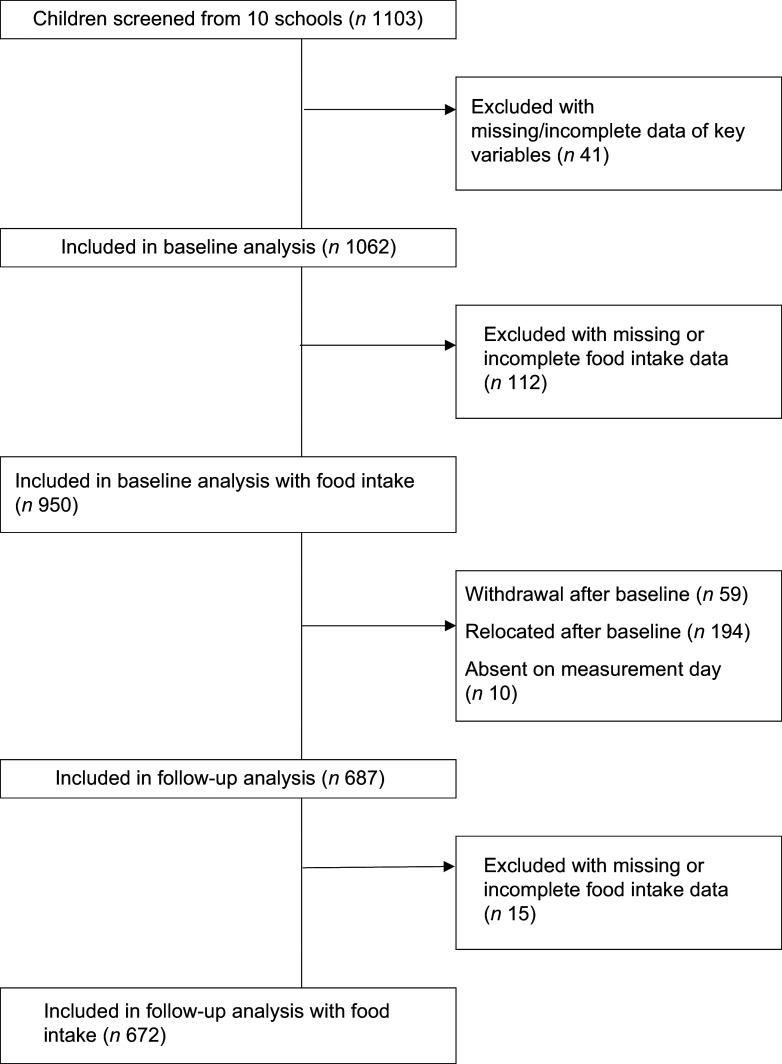
Participant flow diagram for this longitudinal study.

Male and female children aged 5–9 years from all ethnic groups were recruited from the schools. After their parents signed informed consent, 1103 children were available to participate in the study at baseline. Strict exclusion criteria were not applied, but children who showed symptoms of minor ailments on the day of measurement were not included. Each child younger than 7 years signed an informed assent form, while those 7 years and older signed an informed consent form before data collection. A total of 950 children were included at baseline, after excluding children with missing or incomplete data for main variables of interest. At follow-up, data from 687 of these children were collected. Complete baseline and endline data for this study were available for 672 children. Figure 1 shows the participant flow diagram for this longitudinal study.

At baseline, data were collected at each school according to the data collection programme. A General Health and Demographics Questionnaire and the food intake questionnaire were delivered to parents to complete and return to the research team on the day of data collection, as reported elsewhere^(14)^. The General Health and Demographics Questionnaire included data on the education level of the parents, employment, type of housing, household income and self-reported health status^(14)^.

Postgraduate health sciences students performed anthropometric measures according to the International Society for the Advancement of Kinanthropometry guidelines^(17)^. Body weight without shoes and in light clothing was measured on a Seca 813 digital scale to the nearest 0·1 kg. Height was measured to the nearest 0·1 cm with a Seca 213 stadiometer (Birmingham, United Kingdom). All measurements were repeated at least twice, and the average of the two closest measurements was used in data analysis. BMI was calculated by dividing weight in kilograms by height in metre squared (kg/m^2^). The WHO AnthroPlus 2006 software was used to calculate the BMI-for-age z-scores (BAZ)^(18,19)^ and categorise the children as underweight (BAZ < –2), normal weight (BAZ –2 to 1), overweight (BAZ > 1 to 2) and obese (BAZ > 2)^(18)^.

### Food intake questionnaire

Based on evidence of challenges in collecting reliable dietary intake data of children^(20,21)^, a simplified questionnaire was validated to determine the frequency of intakes of specific healthy and unhealthy food groups in South African primary school-age children^(22)^. This unquantified questionnaire was used in the present study and includes four groups of healthy foods (fruits, vegetables, milk, meat/fish/poultry/eggs) and six groups of unhealthy foods, namely, hot SSB, cold SSB, sweets, salty snacks, cakes and fast foods^(22)^. The questionnaire was based on the global school-based student health survey (GSHS) questionnaire^(23)^ and further developed based on questionnaires previously used in Africa, Australia and the USA^(7,24–27)^. The final questionnaire was based on the questionnaires used in studies from South Africa^(24,28,29)^ and included foods usually eaten by South African schoolchildren^(28,30)^. Four groups of healthy foods, namely, fruits, vegetables, milk, meat/fish/poultry/eggs, and six groups of unhealthy foods, namely, hot beverages (tea and coffee) with sugar, cold SSB, sweets, salty snacks, cakes and fast foods, were included. Cold SSB were defined as cold drinks with added sugar, such as sugar-sweetened carbonated drinks, flavoured juice, fruit drinks and sport drinks. The five different responses for frequency of intake were never, 1–2 d, 3–4 d, 5–6 d or 7 d per week. Healthy foods (fruits, vegetables, milk, meat/ fish/ poultry/ eggs) are good sources of essential nutrients for child growth and health^(24)^. Foods that provide energy, sugar, salt and fats but are low in essential nutrients were defined as unhealthy foods^(31)^. The same measurements of the children were repeated in 2021–2022, after 4 years of follow-up since 2017–2018. The health promotion levy on cold SSB at the point of sale in South Africa was implemented in 2018 after the completion of baseline measurements.

### Statistical analysis

Descriptive data of demographic information (age, education of caregivers, household income and home language), anthropometric information and frequency of intakes from food groups are presented. The distribution of data was checked for normality using the Kolmogorov–Smirnov test and QQ plots. Descriptive statistics were reported using median and interquartile ranges for non-normal data and means and sd for normally distributed data, and for categorical characteristics, counts and percentages are presented. The five different responses of frequency of intake, namely, never, 1–2 d, 3–4 d, 5–6 d, or 7 d per week were coded as 0, 1, 3, 5 and 7. Missing data were not imputed, and tests were performed for cases with complete data for each test. The baseline age and school quintile distributions of all children at baseline were compared with the same variables of the group lost to follow-up, using the McNemar test.

A random intercept generalised linear mixed model analysis was applied to investigate the time effect of the weekly intake of the foods adjusting for baseline age and sex of the children. The model was based on the negative binomial distribution with a logarithmic link to account for the potential overdispersion of the outcomes; exponentiated least square means at baseline and at the end of the follow-up were reported and compared by a Wald *t* test^(32)^. First, the negative binomial distribution has been chosen to account for potential overdispersion. Second, a random intercept effect was chosen to better consider individual variability. The least square means have been performed by time points and retro-transformed data. The *P* value for the time effect is reported by means of the Wald test for the hypothesis of parameter estimates difference between the time points. The exponential transformation has been applied and interpreted as a relative risk. The same model with interaction terms between the household income, parental education, child weight status and age and time (factors) has been applied to investigate the effect of the above factors for those foods with a significant change over the observational time. All statistical tests were two tailed with a type-I error rate of 5 % as the threshold for statistical significance. Analysis was performed using SPSS version 29 for Windows (SPSS) and SAS version 9.04 (PROC GLIMMIX).

## Results

**Fig. 2:**
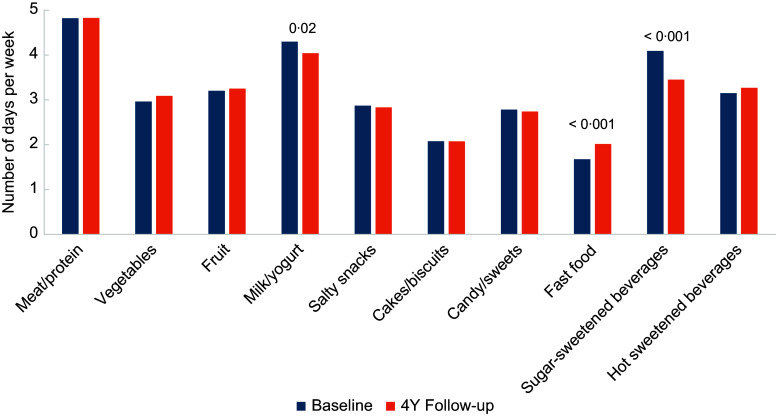
Least squares estimates from mixed model analysis. Bars portray the frequency of intakes (number of days/week) from healthy and unhealthy food groups at baseline and at the end of the study.

The basic sociodemographic and anthropometric characteristics of the participants are presented in Table 1. A comparison of the baseline age and quintile distributions of all children at baseline with the same variables of the group lost to follow-up revealed no statistically significant differences between the variables, although relatively more children from quintile 5 (31·1 % *v.* 26·2 %) were lost to follow-up than from quintile 3 (29·2 % *v.* 32·8 %), compared with the baseline proportions. The weekly frequencies of intakes from the different food groups are shown in Fig. 2. The weekly frequency of intake from most food groups did not change from baseline to follow-up. However, significant changes over 4 years were observed for the weekly intake of milk and yogurt that decreased from 4·3 to 4·0 times per week (*P* = 0·02). Moreover, we reported that the weekly intake of fast food increased from 1·7 to 2 times per week (*P* < 0·001), while the weekly consumption of SSB decreased from 4·1 to 3·1 times per week (*P* < 0·001).

**Table 1.** Baseline descriptive characteristics of 5- to 9-year-old children (*n* 950)

Characteristic	Mean/frequency	sd/%
Age, years	7·4	0·92
Female sex, *n* (%)	507	53·4
Race:		
Black, *n* (%)	520	54·7
White, *n* (%)	409	43·1
Asian and mixed race, *n* (%)	20	2·1
School quintile based on employment rate and literacy in the catchment living area: *n* (%)		
Quintile 3, no-school fee schools, government funded	312	32·8
Quintile 4, fee-paying schools, second highest income quintile	388	40·8
Quintile 5, fee-paying schools, highest income quintile	249	26·2
Anthropometric data:		
BMI-for-age z-score	0·03	1·1
BMI-for-age z-score categories		
Underweight, *n* (%)	36	3·8
Lean, *n* (%)	730	76·8
Overweight, *n* (%)	142	14·9
Obese, *n* (%)	42	4·4

Values are presented as mean (sd) or number of participants and percentage.

**Fig. 3:**
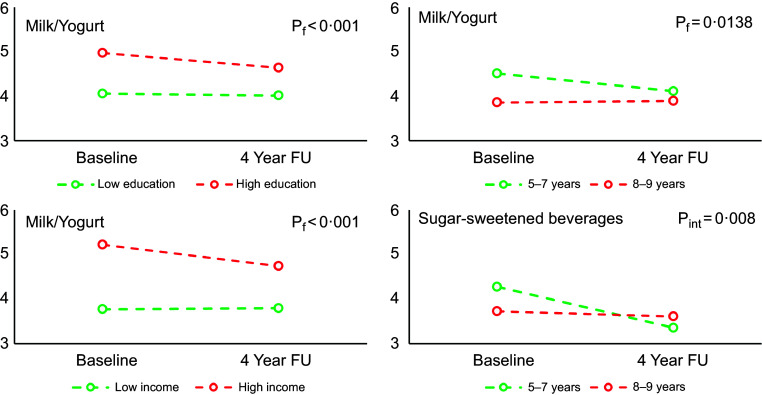
Least squares estimates from mixed model analysis of frequency of SSB intakes between high *v.* low income, parental high *v.* low educational status, overweight-obese *v.* normal weight and age categories from baseline to end of the study (number of days/week). *P*_f_ represents the *P* value for the factor under analysis, and *P*_int_ represents the *P* value for the interaction between the factor and the time.

Figure 3 shows the significant interactions between time and the different age (5–7 years *v.* 8–9 years old), household income (low *v.* high) and parental education (low *v.* high) categories. A statistically significant interaction term age group by time factor was observed for the changes in weekly frequency of intakes of SSB. These results indicate that baseline age, but neither parental education nor household income or weight categories were associated with 4-year changes in intake of SSB. No change in frequency of intake was observed among children 8–9 years old at baseline, but the frequency of intake decreased significantly among younger children. Moreover, a statistically significant effect of income, education and age group was observed for milk-yogurt intake. The frequency of intakes from this group was higher among children from higher-income households and with higher parental education, as well as among younger children at baseline. No significant interactions between time and weight status (normal weight *v.* overweight/obese) were found.

## Discussion

The main findings of this study were the decreased frequency of intake from SSB by South African children, 5–9 years old, over a follow-up period of 4 years. The decreased frequency of SSB intake followed the implementation of the health promotion levy in 2018 after baseline, in line with reports of national decreases in SSB sales in South Africa since 2017. The decreased frequency of SSB intakes showed an interaction with baseline age, with a decreased frequency of intakes more evident among the youngest children. This may be an indication that the availability of SSB within households decreased over the 4 years of follow-up.

We explored baseline variables that could be driving these changes in frequencies of intakes from the SSB group. Age was considered because as children grow older, parents may offer more or less of particular foods, or children may select more frequent intakes from some foods based on peer pressure, media exposure or change in their social habits^(24)^. The results confirm a decreased frequency of SSB intakes among the younger age group, who may be less exposed to peer pressure and more dependent on their parents’ decision to purchase SSB. This observation may reflect the reduction in sales to households after the levy has been implemented^(10)^. BAZ may be a potential driver of these changes in frequencies of intakes from the SSB group. Parents of children living with overweight may have received advice from health professionals to offer less SSB, fast food and salty snacks to their children^(31)^. There were, however, no differences in the changes in SSB intakes of children of these different categories. There were also no differences in the changes in intakes from the fast food and milk groups of children from different weight categories.

Educational status of caregivers may also play a role in changing eating patterns of children. Parents from a higher educational level may be more prone to offer healthy food choices at home or when eating out. Educational status is generally associated with household income. Families with a higher income have access to more expensive foods, such as animal source protein foods, milk, yogurt and fruits. These families are also less affected by food price increases, such as levies on particular food groups^(10)^. In the present study, no differences in SSB intakes between children from different income or educational status categories were found. There were however effects of parental education, household income and age on the decreased frequencies of intakes from the milk group. Among children from low-income households and older children frequency of milk intakes remained low across the 4 years of follow-up. After 4 years frequency of milk intakes was still higher among children from high-income households, but the decreased frequency of intakes over time probably reflects the trend of lower milk intakes among children with increasing age^(30)^.

The Health Promotion Levy on SSB was introduced in 2018 and implemented after baseline measurements have been completed. A recent South African study showed a decrease in mean sugar content from taxable SSB purchases from 16·2 to 10·6 g/capita per d 1 year after implementation. Mean volumes of taxable SSB purchases decreased from 519 to 443 ml/capita per d over the same period. These decreases were accompanied by a small increase in the purchases of non-taxable cold beverages, from 283 to 313 ml/capita per d post-implementation. The frequency of intakes of non-taxable drinks was not assessed in the present study. Before the levy was implemented, lower socio-economic status households purchased larger amounts of taxable SSB than higher socio-economic status households, but there was a bigger reduction in sales to lower socio-economic status households after the levy was implemented^(10)^. In the present study, no differences in the frequency of SSB intakes between income groups were apparent at either baseline or end. The reason for this lack of difference may be that children from the lowest income group were not included in the present study. The expected social gradient in response to the implementation of the health promotion levy was not demonstrated in this study, probably due to exclusion of children from quintiles 1–2 or due to the relatively smaller sample size from two municipalities, compared with the country-wide study with a much larger sample of 113 653 households across South Africa^(10)^.

According to the South African food-based dietary guidelines, children should consume vegetables and fruit daily and use fats, salt and sugar sparingly^(33)^. In line with the results of the present study, insufficient intakes of fruit and vegetables have been reported frequently among South African children, with possible adverse health implications^(34)^. Furthermore, frequent intakes of unhealthy snack foods and SSB have been reported across household income groups^(28,34,35)^. The finding that the frequency of consumption of fast foods increased significantly between baseline and follow-up is of concern. There are currently few national strategies in South Africa to combat the high intakes of fast foods and other processed foods with a high content of fat and salt. The South African government implemented strategies to improve school-age child nutrition, including the Integrated School Health Policy and the National School Nutrition Programme. Most of these measures and policies aim at improving the micronutrient nutritional status of children, with no clear focus on the restriction of unhealthy foods^(36)^.

We further explored replacement of cold SSB intake by other food groups. No significant differences between baseline and follow-up frequencies of hot SSB, or fruit intakes were found, indicating that a decreased frequency of cold SSB intake was not associated with increased frequencies of other drinks or fruit intakes. On the contrary, the frequency of milk intake also decreased at follow-up. It is unknown if the SSB were replaced by the low kJ beverages or water because these items were not included in the food questionnaire.

The possibility that being included in this study may have had an impact on the health behaviour in households of the participants should be considered. After baseline, basic health information pamphlets were handed out to children. The information included healthy food choices that may have had an impact on the frequency of SSB intake of the children. However, after follow-up, there were no significant increases in the frequency of intakes of healthy foods, but there was an increase in the intakes of fast foods. It is also possible that fast-food meals could have included SSB, but that was not reported separately. It appears that the frequency of SSB intake between meals on its own decreased, which may be an indication that a smaller volume of SSB were available in households at follow-up, compared with baseline. This finding is in line with the South African study that found that mean volumes of taxable SSB purchases decreased from 519 to 443 ml/capita per d 1 year after implementation of the Health Promotion Levy on SSB^(10)^.

### Limitations

The children included in this study attended schools from the South Africa quintile groups 3–5, indicating medium to high employment rate and literacy of the communities where the schools are located. Quintiles 1 and 2 represent the poorest schools^(16)^, and therefore, participants from the lowest socio-economic status groups were not part of this study. The results may therefore not apply to children from the lowest socio-economic status groups in South Africa. Furthermore, we do not know if the SSB were replaced by the low kJ beverages or water because this question was not included in the questionnaire. Despite these limitations, the strength of the study was that it was the first longitudinal study of intakes from healthy and unhealthy food groups in a relatively large sample of primary school-aged South African children over a 4-year period, after the implementation of a health promotion levy on SSB.

### Summary and conclusion

The implementation of health policies, such as the health promotion levy in South Africa, may have a significant impact on improving health promotion and disease prevention. This is evidenced by the observed decrease in the frequency of SSB intake across various socio-economic and BAZ categories following the implementation of the health promotion levy in South Africa. This suggests that such policies can influence behavioural changes in the population, leading to healthier lifestyle choices and potentially reducing the risk of diseases associated with high SSB consumption. Therefore, the health promotion levy serves as a beneficial strategy in public health policy to promote healthier dietary habits and prevent disease. Future strategies to combat the high intakes of fast foods and other processed foods with a high content of fat and salt may have a similar impact.

## Supporting information

Kruger et al. supplementary material Kruger et al. supplementary material

## Supplementary material

For supplementary material accompanying this paper, visit https://doi.org/10.1017/S1368980024002623.
