# Supplementary material for: Decreased frequency of sugar-sweetened beverages intake among young children following the implementation of the health promotion levy in South Africa
Source: Public Health Nutr. 2025 Jan 7;28(1):e23. doi: 10.1017/S1368980024002623 (PMC11822614; doi:10.1017/S1368980024002623)
Supplement: Kruger et al. supplementary material [file S1368980024002623sup001.docx]

Supplementary table 1. Summary tables of the association of food intake at the baseline and the end of the study.

|  | ^a^β (std) | ^b^Exp(β) (95% CI) | ^c^Pvalue | ^d^Pseudo R^2^ | Time | ^e^Estimate by time |
| --- | --- | --- | --- | --- | --- | --- |
| Meat/protein | 0.001 (0.021) | 1.00 (0.96; 1.04) | 0.9490 | 0.0011 | Baseline | 4.82 (4.62; 5.03) |
|  |  |  |  |  | End FU | 4.83 (4.61; 5.06) |
| Vegetables | 0.041 (0.030) | 1.04 (0.98; 1.11) | 0.1669 | 0.0057 | Baseline | 2.96 (2.85; 3.08) |
|  |  |  |  |  | End FU | 3.09 (2.95; 3.23) |
| Fruit | 0.015 (0.031) | 1.01 (0.96; 1.08) | 0.6280 | 0.0088 | Baseline | 3.20 (3.08; 3.33) |
|  |  |  |  |  | End FU | 3.25 (3.10; 3.41) |
| Milk/Yogurt | -0.062 (0.026) | 0.94 (0.89; 0.99) | 0.0196 | 0.0074 | Baseline | 4.30 (2.82; 6.56) |
|  |  |  |  |  | End FU | 4.04 (2.65; 6.17) |
| Salty snacks | -0.014 (0.035) | 0.99 (0.92; 1.06) | 0.6832 | 0.0049 | Baseline | 2.87 (2.24; 3.68) |
|  |  |  |  |  | End FU | 2.83 (2.21; 3.64) |
| Cakes/biscuits | -0.002 (0.041) | 1.00 (0.92; 1.08) | 0.9671 | 0.0007 | Baseline | 2.08 (1.97; 2.19) |
|  |  |  |  |  | End FU | 2.07 (1.95; 2.21) |
| Candy/sweets | -0.016 (0.035) | 0.98 (0.92; 1.05) | 0.6526 | 0.0037 | Baseline | 2.78 (2.67; 2.91) |
|  |  |  |  |  | End FU | 2.74 (2.60; 2.89) |
| Fast food | 0.184 (0.042) | 1.20 (1.11; 1.30) | <.0001 | 0.0128 | Baseline | 1.68 (1.59; 1.77) |
|  |  |  |  |  | End FU | 2.02 (1.90; 2.15) |
| Sugar sweetened beverages | -0.170 (0.030) | 0.84 (0.80; 0.89) | <.0001 | 0.0207 | Baseline | 4.09 (3.95; 4.24) |
|  |  |  |  |  | End FU | 3.45 (3.30; 3.61) |
| Hot sweetened beverages | 0.038 (0.040) | 1.04 (0.96; 1.12) | 0.3521 | 0.0028 | Baseline | 3.15 (2.98; 3.32) |
|  |  |  |  |  | End FU | 3.27 (3.09; 3.46) |
| Notes. **a**: Coefficient of the time effect and standard error, **b**: Exponential of the coefficient of the time effect and 95% CI representing the relative risk of one unit increase of the frequency of consumption for that specific food, **c**: P-Value for the coefficient corresponding to a significant time effect, **d**: Pseudo R2 of the model, **e**: exponentiated least square mean representing the frequency of consumption by time point | | | | | | |
